# A rare myoepithelioma of the sinonasal cavity: case report

**DOI:** 10.1186/1757-1626-1-29

**Published:** 2008-07-11

**Authors:** Suhail I Sayed, Rehan A Kazi, Mohan V Jagade, Rajan S Palav, Vinod V Shinde, Prashant V Pawar

**Affiliations:** 1 Department of ENT and Head and Neck surgery, Grants medical college and Sir JJ group of hospitals, Mumbai 400008, India

## Abstract

Myoepithelioma is a rare benign neoplasm. Pure accounting for less than 1% of all salivary gland tumors. Only three cases of sinonasal myoepithelioma have been reported in the literature. Diagnosis of myoepithelioma through light microscopy is possible and immunohistochemistry is done to facilitate the diagnosis. The lesion is so rare that there are no specific indications/guidelines for its treatment. We report to you a rare case of sinonasal myoepithelioma in a 57 year old Asian female.

Myoepitheliomas are rare tumours that account for only about 1% of all salivary gland tumors. Most are benign, but some can be malignant. Only three cases of sinonasal myoepithelioma have been reported in the literature so far.

## Case history

**Figure 1:**
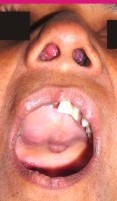
Pinkish polypoidal soft tissue seen in the nasal cavity with smooth bulging of the hard palate.

**Figure 2:**
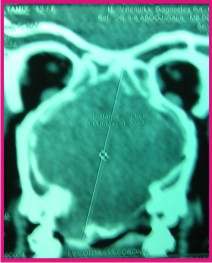
Contrast enhanced CT scan (coronal view) showing an oval lobulated iso-dense mass measuring about 7.0 × 5.0 cm confined to the nasal vault and the ethmoids.

A 57 year old female presented to the out-patient department (OPD) of a leading multispeciality teaching hospital with a 3 year history of progressively increasing right sided nasal blockage, a change in voice and a bulge over the hard palate. No history of epistaxis was given. On nasal examination, there was a broadening of the nasal bridge and a large pinkish red polypoidal mass was seen in the right nasal cavity. Oral cavity examination showed a smooth bulge over the hard palate mostly confined on the right side (Figure 1). CT scan done showed an oval lobulated iso-dense patchily enhancing mass measuring about 7.0 × 5.0 cm with its epicentre in nasal vault and the ethmoids (Figure 2).

An incisional biopsy was performed as an OPD procedure and this revealed loose clusters of plasmacytoid (hyaline) myoepithelial cells (40×).

**Figure 3:**
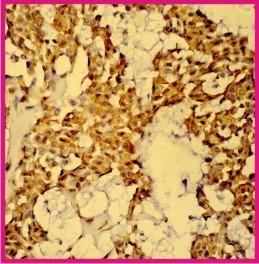
**Tumour is composed of small and/or medium spindle shaped cells, differently interlaced, with eosinophilic cytoplasm, that occur in sheets or swirls and have round to oval nuclei with finely dispersed chromatin.** Stroma is myxomatous. Myoepithelial cells stain strongly positive for Calponin (40×).

Subsequently, following a multidisciplinary meeting, the patient underwent a sublabial surgical approach for resection of the tumor. On table the sessile, encapsulated, pinkish red and firm tumor was occupying whole of the nasal cavity with the septum pushed to the left side. Histopathological examination of the specimen revealed loose clusters of plasmacytoid (hyaline) small and/or medium sized spindle shaped cells, differently interlaced, with eosinophilic cytoplasm, occurring in sheets or swirls; the nuclei were predominantly round to ovoid in shape, often eccentric, with finely dispersed chromatin and low mitotic activity. Further Immunohistochemical studies revealed positivity to Calponin (Figure 3). These results were consistent with the rare diagnosis of a sinonasal myoepithelioma. Patient had no recurrence during a follow-up period of 2 years.

## Discussion

Myoepitheliomas are rare tumors that account for only about 1% of all salivary gland tumors. Most are benign, but some can be malignant. In 1943 Sheldon first described myoepithelioma in the literature and then later in 1975 Stromeyer et al reported the first documented case of a malignant myoepithelioma. [1]

Myoepithelial cells are present in many secretory organs and have a dual epithelial and smooth muscle phenotype. Salivary glands have these cells in their acini and intercalated ducts. These cells form an important component in many salivary gland neoplasms. The tumor occurs over a wide age range with a median of 53 years and has no sex predilection. It usually presents as a painless, slow growing mass of benign nature, but sometimes it may be locally aggressive. The parotid gland is preferentially involved as compared to the other salivary glands. [2]

Only three cases of sinonasal myoepithelioma have been reported in the literature so far. [3-5] The CT imaging appearance of myoepithelioma (a well-defined enhancing mass) is not specific. [5]

Macroscopically benign myoepitheliomas are well circumscribed and encapsulated masses with a smooth external appearance and a white, tan or gray cut surface but there are no distinctive features. Microscopically several growth patterns occur: solid, the most common, myxoid (pleomorphic adenoma like), reticular and mixed. Cells can vary in histological appearance being spindle-shaped, plasmacytoid, and epitheloid; occasionally, epitheloid cells have clear cytoplasm. The different cellular compositions have no correlation to prognosis. [6,7]

Although myoepithelioma has been defined as a neoplasm exclusively composed of myoepithelial cells, many authors have introduced a less rigid definition to include tumors with a small number of ductal cells (less than 10% of the surface area of the tumor). Probably myoepitheliomas constitute one end of a biological spectrum that includes pleomorphic adenoma in the middle and non-membranous basal cell adenomas at the other end. [6,7]

Even though the entity is rare and the histopathology might be quite varied, diagnosis of myoepithelioma by light microscopy is possible and to facilitate the diagnosis immunohistochemistry is also performed. Almost all cases of myoepithelioma are strongly positive for Calponin, S-100 protein and the tumor cells also display varying degrees of immunoreactivity for cytokeratins, Glial fibrillary acidic protein (GFAP), myosin, actin, vimentin and carcinoembryonic antigen (CEA).

The most ultrastuctural component of myoepithelial cells are the myofilaments which may appear in well-aligned bundles or as disordered arrays, desmosomes are also commonly present. [6]

The differential diagnosis of myoepitheliomas includes pleomorphic adenoma and other mesenchymal neoplasms, nasal fibrosarcoma, fibromatosis of the sinonasal tract, neoplasms of smooth muscles, sinonasal myxoma and embryonal rhabdomyosarcoma. Malignant myoepitheliomas have been described that infiltrate the surrounding tissue and even metastasize. Such myoepitheliomas can arise either de-novo or develop ex pleomorphic adenoma or ex benign myoepithelioma. [8,9]

Classical myoepitheliomas are not more aggressive than benign mixed tumors as once thought. Instead, they are essentially the same benign tumor at a different point along a histological continuum. [6,10]

The treatment of a myoepithelioma is generally considered as complete surgical excision. [1]

## Consent

Written informed consent was obtained from the patient for publication of this Case report and any accompanying images. A copy of the written consent is available for review by the Editor-in-Chief of this journal.

## Competing interests

The authors declare that they have no competing interests.

## Authors' contributions

SS The lead author involved in carrying out the literature search, study design and writing the case report. PP, RP, VS assisted with writing the paper, supervising and designing the study. RK, MJ supervised the management of the case and participated in its design and approval. All authors have been involved in approving the final manuscript.
